# Ampelopsin induces apoptosis by regulating multiple c-Myc/S-phase kinase-associated protein 2/F-box and WD repeat-containing protein 7/histone deacetylase 2 pathways in human lung adenocarcinoma cells

**DOI:** 10.3892/mmr.2014.2733

**Published:** 2014-10-21

**Authors:** XIN-MEI CHEN, XIAN-BIAO XIE, QING ZHAO, FANG WANG, YANG BAI, JUN-QIANG YIN, HONG JIANG, XIAO-LIN XIE, QIANG JIA, GANG HUANG

**Affiliations:** 1 Department of Biochemistry, School of Basic Science, Guangzhou Medical University, Guangzhou, Guangdong 510182, P.R. China; 2 Department of Musculoskeletal Oncology, The First Affiliated Hospital of Sun Yat-Sen University, Guangzhou, Guangdong 510080, P.R. China; 3 Institute of Biology, Guizhou Academy of Sciences, Guiyang, Guizhou 550009, P.R. China; 4 Guangzhou Institute of Biomedicine and Health, Chinese Academy of Sciences, Guangzhou, Guangdong 510230, P.R. China

**Keywords:** lung cancer, ampelopsin, c-Myc, S-phase kinase-associated protein 2, F-box/WD repeat-containing protein 7

## Abstract

Ampelopsin (AMP), a plant flavonoid, has been reported to inhibit cell growth and/or induce apoptosis in various types of tumor. The aim of the present study was to assess the apoptosis-inducing activity of AMP in A549 human lung adenocarcinoma epithelial cells and the associated underlying mechanism. A549 cells were incubated with different concentrations of AMP in culture medium. Cell growth and apoptosis were evaluated by MTT assay and Annexin V/propidium iodide double staining and flow cytometry, respectively. In addition, western blotting and reverse transcription quantitative polymerase chain reaction analysis were used to examine the time-dependent changes in protein expression. Certain changes in apoptotic protein expression were detected following exposure to AMP, including X-linked inhibitor of apoptosis protein release, reduced B-cell lymphoma 2, myeloid cell leukemia 1 and survivin expression levels, increased Bcl-2-associated X protein expression levels and cleaved-poly ADP ribose polymerase expression. The results revealed that AMP was a potent inhibitor of A549 cell proliferation. The c-Myc/S-phase kinase-associated protein 2 (Skp2) and histone deacetylase (HDAC)1/2 pathways were found to exert an important role in AMP-induced A549 cell apoptosis, as increased levels of c-Myc mRNA and reduced levels of c-Myc/Skp2 and HDAC1 and 2 proteins following AMP treatment were observed. The levels of F-box and WD repeat-containing protein 7α (Fbw7α), Fbw7β, Fbw7γ, phosphorylated-(p−)c-Myc (Thr58) and glycogen synthase kinase 3β (GSK3β) proteins involved in c-Myc ubiquitin-dependent degradation were also analyzed. Following exposure to AMP, the expression levels of Fbw7α, Fbw7γ and GSK3β were reduced and p-c-Myc (Thr58) expression levels were increased. The results suggest that AMP exerts an anticancer effect, which is associated with the degradation of c-Myc, Skp2 and HDAC1 and 2. The ability of AMP to induce apoptosis independently of Fbwα and Fbw7γ suggests a possible use in drug-resistant cancer associated with Fbw7 deficiency. Understanding the exact underlying mechanism requires further investigation of the association between c-Myc and Fbw7α/γ reversal, and analysis of whether Thr58 phosphorylation of c-Myc is dependent on GSK3β.

## Introduction

Despite multiple clinical trials aimed at improving patient survival rates, lung cancer is the second leading cause of cancer-related mortality worldwide following breast cancer. Among all types of pulmonary cancer, ~85% of cases are diagnosed, commonly at advanced stages, as non-small cell lung cancer (NSCLC) (1). Lung adenocarcinoma is the predominant NSCLC histological subtype, which accounts for 20–30% of primary lung cancer cases among patients <45 years of age, regardless of smoking history (2). The majority of patients with NSCLC exhibit advanced disease, thus are unsuitable for surgery; therefore, chemotherapy remains the cornerstone of treatment.

Histone deacetylases (HDACs) are enzymes that remove histone acetylation markers, resulting in compaction of the chromatin structure and transcriptional repression (3). In addition to histones, HDACs have various nonhistone protein substrates involved in the regulation of gene expression, cell proliferation, cell migration, apoptosis and angiogenesis (4). A number of naturally occurring and synthetic HDAC inhibitors have been shown in preclinical studies to exert potent anticancer activity (5).

HDAC1 and 2 are similar enzymes that belong to the class I HDAC family of molecules. *In vivo*, HDAC1 and 2 cooperate within a complex of proteins including Sin3, nucleosome remodeling and deacetylating, and repressor element-1 silencing transcription factor corepressor 1 (6). In addition to functions exerted through these complexes, HDAC1 and 2 also bind directly to DNA-binding proteins, including Yin and Yang 1, retinoblastoma binding protein-1 and specificity protein 1 (6).

c-Myc, as a transcription factor, is responsible for the regulation of multiple genes associated with cellular activities including cellular proliferation, growth, apoptosis and differentiation (7). Due to the extensive functions of c-Myc, it is considered to be a potent oncogene. In addition, downregulated c-Myc expression has been observed in ~70% of all types of human tumor (8). The expression of c-Myc is controlled at a number of different levels, including gene transcription, mRNA stability and through the post-translational control of protein stability (9–11). Post-translational regulation of c-Myc is mediated by S-phase kinase-associated protein 2 (Skp2) and F-box and WD repeat-containing protein 7 (Fbw7) (12), two recognition subunits of the Skp1/Cullin/F-box protein complex-type E3 ligase that have the ability to recognize the substrates responsible for proteasomal degradation.

Skp2 overexpression is frequently observed in human cancer specimens and has been suggested to be an oncogene. With the exception of c-Myc, Skp2 is reported to recognize cyclin-dependent kinase inhibitors and tumor suppressor proteins such as p27 Kip1, p57 Kip2, p130 and Tob1 (13–16). Fbw7 has frequently been found to be inactivated by mutation, deletion or promoter hypermethylation in multiple types of neoplasm, including breast cancer (17,18), colon cancer (19,20) and leukemia (21).

The involvement of Fbw7 deficiency in human cancer drug resistance has been recently detected (22,23). In the regulation of c-Myc, c-Myc phosphorylation at Thr58 is required for Fbw7-mediated proteasomal degradation. Glycogen synthase kinase 3β (GSK3β) is the only known kinase that phosphorylates c-Myc at Thr58 (24). In contrast to Fbw7, Skp2-mediated ubiquitylation of c-Myc does not require its phosphorylation (25). Fbw7 is expressed as three isoforms in humans, designated α, β and γ, located in the nucleus, cytoplasm and nucleolus, respectively (26,27).

**Figure 1:**
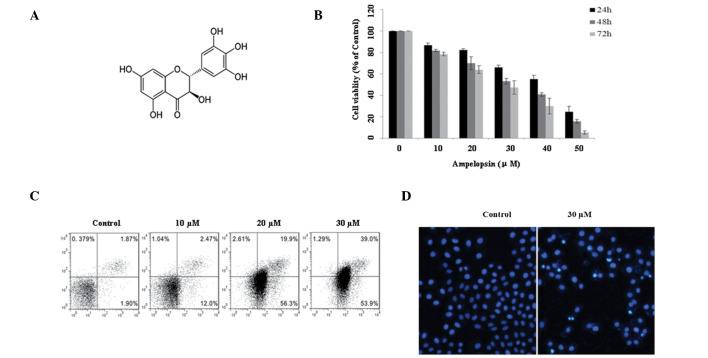
Ampelopsin exerts potent anti-A549 cell activity *in vitro*. (A) Chemical structure of ampelopsin. (B) A549 cell viability was analyzed using the MTT assay. The cells were incubated with ampelopsin for 24, 48 or 72 h. The bars indicate the mean value ± standard error of the mean (n=3). (C) Apoptosis assessment using Annexin V/PI staining of A549 cells. The cells were incubated with ampelopsin at concentrations of 0, 10, 20 or 30 μM, for 48 h. Early apoptotic cells were Annexin V-positive and PI-negative, and late apoptotic and dead cells were Annexin V-positive and PI-positive. (D) Nuclei stained by DAPI and observed by fluorescence microscope. Apoptotic cells are indicated by arrows. PI, propidium iodide.

Ampelopsin [AMP; (2R,3R)-3,5,7-trihydroxy-2-(3,4,5-trihydroxyphenyl)-2,3-dihydrochromen-4-one; Fig. 1A], is a naturally occurring flavonoid isolated from the plant species *Ampelopsis grossedentata* (Hand.-Mazz) W.T. Wang. The anticancer activity of AMP has been reported in various human cell lines, including bladder carcinoma (28), melanoma (29), GLC-82 lung cancer (30), hepatocellular carcinoma (31), K562/ADR leukemia (32) and prostate cancer cells (33). AMP activity is mediated by the induction of apoptosis and cell differentiation, which is regulated by various genes and proteins (34). In the present study, the effect of AMP upon cell proliferation and apoptosis in the A549 human lung adenocarcinoma epithelial cell line was assessed. In addition, the regulatory effects and underlying functions of the c-Myc/Skp2/Fbw7 and HDAC1/2 pathways involved in the apoptotic effect were investigated.

## Materials and methods

### Reagents and antibodies

AMP (purity >98%) was supplied by the Institute of Biology at Guizhou Academy of Sciences (Guiyang, China). The following antibodies were used: Fbw7 (cell division control protein 4, H-300) and phosphorylated-(p−)c-Myc (Thr 58) were provided by Santa Cruz Biotechnology, Inc. (Santa Cruz, CA, USA); c-Myc and GSK3β were bought from Proteintech Group, Inc. (Chicago, IL, USA); HDAC1 and 2, Skp2, survivin, B-cell lymphoma 2 (Bcl-2) and β-actin were obtained from Wuhan Boster Biological Technology, Ltd. (Wuhan, China); myeloid cell leukemia 1 (Mcl-1) and X-linked inhibitor of apoptosis protein (XIAP) were provided by Bioss (Beijing, China); and poly ADP ribose polymerase (PARP) was purchased from Sino Biological Inc. (Beijing, China).

### Cell culture

A549 human pulmonary adenocarcinoma cells were provided by the Cell Bank of the Animal Experiment Center, North School Region, Sun Yat-Sen University (Guangzhou, China). The A549 cells were cultured in RPMI-1640 medium containing 10% fetal bovine serum (Hangzhou Sijiqing Biological Engineering Materials Co., Ltd., Hangzhou, China) at 37°C and 5% CO_2_.

### MTT assay

To measure cell viability, A549 cells harvested with trypsin were seeded onto 96-well plates at a density of 1×10^4^ cells per well. Following overnight incubation, the culture medium was removed and the cells were incubated with different concentrations of AMP in culture medium. After 48 h, MTT was added to each well and incubated at 37°C for an additional 4 h to allow mitochondrial dehydrogenase to convert the MTT into insoluble formazan crystals. The medium was then discarded and 100 μl dimethylsulfoxide (Sigma, St. Louis, MO, USA) was added to each well to dissolve the formazan crystals. The absorption of solubilized formazan was measured at 490 nm using a EL340 microplate reader (Bio-Tek Instruments, Inc., Winooski, VT, USA).

### Nuclear staining

A549 cells were stained using a DAPI staining kit (Nanjing KeyGen Biotech. Co. Ltd., Nanjing, China). Following exposure to graded AMP concentrations for 48 h, the cells were washed and incubated with 1–2 μg/ml DAPI working solution at 37°C for 15 min. The cells were subsequently washed with methanol solution. Buffer A containing 60% glycerol in 10 mM phosphate-buffered saline (PBS; pH 7.6) was added to the suspension. The A549 cells were viewed using an Eclipse Ti Nikon microscope (Nikon Corporation, Tokyo, Japan).

### Mitochondrial membrane potential

A JC-1 fluorescent, lipophilic, cationic probe (Beyotime Co., Shanghai, China), was used to measure the mitochondrial membrane potential (Δψm) of the A549 cells, according to the manufacturer’s instructions. Briefly, the cells exposed to AMP were incubated with 1× JC-1 staining solution for 20 min at 37°C. The cells were then washed twice with JC-1 staining buffer and images were captured with the Eclipse Ti Nikon microscope.

### Apoptosis assay

A549 cells were labeled with fluorescein isothiocyanate (FITC)-labeled Annexin V and propidium iodide (PI) using an Annexin V-FITC apoptosis detection kit (Nanjing KeyGen Biotech. Co. Ltd.) according to the manufacturer’s instructions. Briefly, after 48 h exposure to different AMP concentrations, the cells were washed with cold PBS and then resuspended in 1× binding buffer. Aliquots of 10^5^ cells were mixed with 5 μl Annexin V-FITC and 5 μl PI for 15 min at room temperature in the dark. Fluorescence (530 nm) was detected within 1 h using flow cytometry at a wavelength of 530 nm (FACS Aria; BD Biosciences, Franklin Lakes, NJ, USA).

### Reverse transcription quantitative polymerase chain reaction (RT-qPCR) analysis

RT-qPCR with a SYBR^®^ Green reporter was conducted. The A549 cells exposed to AMP were washed with PBS. Total RNA was purified using RNAiso Plus (Takara, Dalian, China). The resultant RNA was first reverse transcribed to cDNA using a PrimeScript^®^ RT Master Mix kit (Takara). Gene-specific primers were combined with SYBR^®^ Premix Ex Taq™ (Takara) and amplified using an ABI 7500 Real-Time PCR System (Applied Biosystems, Foster City, CA, USA). All qPCR reactions were conducted independently on five samples. The relative mRNA expression levels were calculated using the 2^−ΔΔCt^ method. The primer sequences are described in Table I.

### Western blot analysis

Following washes with PBS, the cells were lysed with radioimmunoprecipitation assay buffer containing 50 mM Tris (pH 7.4), 150 mM NaCl, 1% nonyl phenoxypolyethoxylethanol-40, 0.5% sodium deoxycholate, 0.1% sodium dodecyl sulfate (SDS), sodium orthovanadate, EDTA, sodium fluoride and leupeptin (Beyotime Co.) supplemented with phenylmethylsulfonyl fluoride protease inhibitor (Beyotime Co.). Cytoplasmic proteins were separated using a nuclear and cytoplasmic protein extraction kit (Nanjing KeyGen Biotech. Co. Ltd.). The concentrations of soluble proteins were determined with a bicinchoninic acid protein assay kit (Beyotime Co.). The cell lysates were boiled in loading buffer for 5 min and then separated on a 15% SDS-PAGE gel. The proteins were subsequently transferred to a polyvinylidene difluoride membrane (Millipore, Billerica, MA, USA). The membranes were blocked with Tris-buffered saline-Tween 20 (TBST) containing 5% non-fat milk at room temperature for 1 h, and were incubated with the aforementioned monoclonal primary antibodies (1:1,000) overnight at 4°C. Subsequent to washing three times for 5 min each with 15 ml TBST, the membranes were incubated with the corresponding horseradish peroxidase-conjugated secondary antibodies (1:10,000; Beyotime Co.) for 1 h at room temperature and visualized with enhanced chemiluminescence detection reagents (Beyotime Co.).

### Statistical analysis

Data analysis was performed using SPSS version 17.0 software (SPSS, Inc., Chicago, IL, USA). The results are expressed as the mean ± standard error of the mean from a minimum of three independent experiments. The statistical significance between groups was determined by one-way analysis of variance. P<0.05 was considered to indicate a statistically significant difference.

## Results

### AMP inhibits cell growth and induces cell apoptosis

Cell viability was assessed using an MTT assay together with DAPI staining and flow cytometric analysis, to investigate apoptosis following exposure to different concentrations of AMP. The MTT analysis indicated that AMP inhibited cell growth in a dose-dependent manner (Fig. 1B) and this was confirmed by the results of the flow cytometric analysis using Annexin V-PI (Fig. 1C). As compared with the control group, the early- and late-stage apoptotic rates increased subsequent to exposure to all concentrations of AMP. In the 30 μM AMP group, the total apoptosis rate exceeded 50%. As shown in Fig. 1D, DAPI staining revealed signs of condensed and cleaved nuclei in the cells administered 30 μM AMP, but only clear nuclei with pale blue staining were observed in the control group.

**Figure 2:**
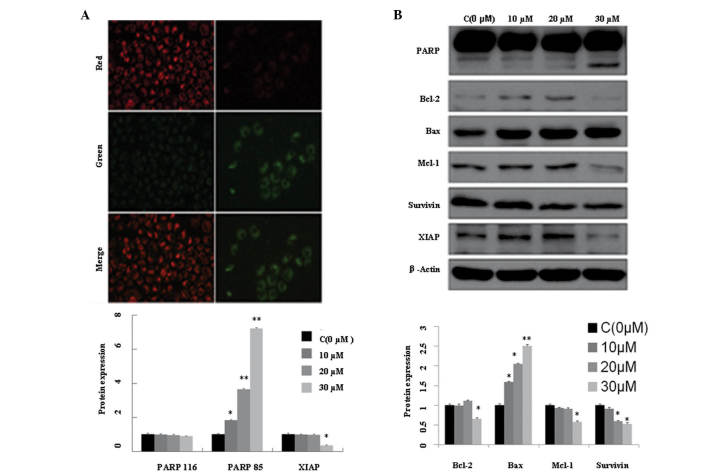
Effects of ampelopsin on mitochondrial membrane potential and apoptotic proteins in A549. (A) Analysis of the mitochondrial membrane potential (ΔΨm) using JC-1 staining following exposure to ampelopsin for 48 h. A fluorescence microscope was used to visualize the results. Mitochondrial depolarization was indicated by an increase in green fluorescence and a reduction in red fluorescence intensity. (B) Bcl-2, Mcl-1, XIAP, survivin and PARP protein levels were assayed by western blotting. ^*^P<0.05 and ^**^P<0.01 vs. the control group. Bcl-2, B-cell lymphoma 2; Mcl-1, myeloid cell leukemia 1; XIAP, X-linked inhibitor of apoptosis protein; PARP, poly ADP ribose polymerase; Bax, Bcl-2-associated X protein.

The results also revealed an association between AMP treatment and a dose-dependent decline in the mitochondrial potential (Δψm), with reduced red fluorescence (JC-1 polymer) and increased green fluorescence (JC-1 monomer), as presented in Fig. 2A. This result may have been due to the downregulation of Mcl-1 and Bcl-2 (Fig. 2B). The upregulation of cleaved fragments from PARP (PARP 85) and the downregulation of XIAP and survivin suggest that the apoptotic process continued following mitochondrial damage.

### AMP downregulates HDAC2 at the mRNA and protein levels

**Figure 3:**
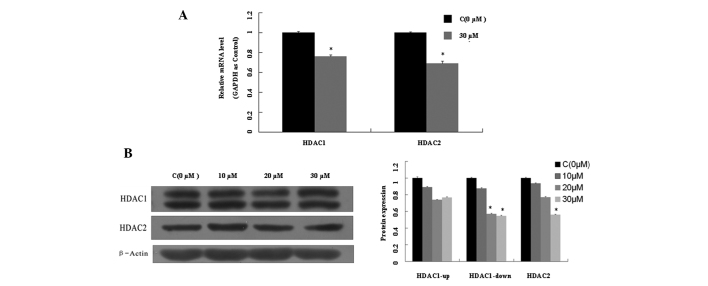
Effects of ampelopsin on HDAC1 and 2 expression in A549 human pulmonary adenocarcinoma cells. (A) The relative HDAC1 and 2 mRNA levels were detected using quantitative polymerase chain reaction, with GAPDH serving as an internal control. The results are expressed as the mean ± standard error of the mean from five independent experiments. (B) HDAC1 and 2 protein levels assayed by western blotting. In these experiments, cells were exposed to 0, 10, 20 and 30 μM ampelopsin for 48 h. β-actin served as an internal control. ^*^P<0.05 and ^**^P<0.01 vs. the control group. HDAC, histone deacetylase.

Significant downregulation of HDAC1 and 2 mRNA following 30 μM AMP treatment, as compared with the control treatment, was detected by qPCR (Fig. 3A). This result is consistent with the finding that HDAC2 protein expression was downregulated in a dose-dependent manner following exposure to different concentrations of AMP for 48 h (Fig. 3B). These changes may result in a subsequent proportional increase in the levels of histone acetylation, therefore enhancing the expression levels of tumor suppressive proteins.

### AMP increases c-Myc mRNA expression levels, but downregulates c-Myc and Skp2 expression at the protein level

**Figure 4:**
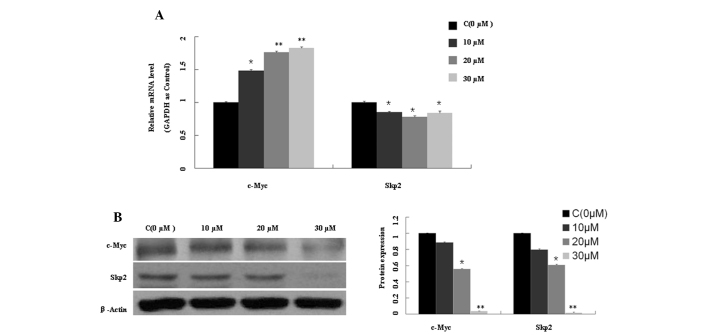
Effects of ampelopsin on c-Myc and Skp2 expression levels in A549 human pulmonary adenocarcinoma cells. (A) The relative c-Myc and Skp2 mRNA levels were detected using quantitative polymerase chain reaction with GAPDH as an internal control. The results are expressed as the mean ± standard error of the mean from five independent experiments. (B) c-Myc and Skp2 protein levels were assayed by western blotting. β-actin served as an internal control. In these experiments, the A549 cells were exposed to 0, 10, 20 and 30 μM ampelopsin for 48 h. ^*^P<0.05 and ^**^P<0.01 vs. the control group. Skp2, S-phase kinase-associated protein 2.

As shown in Fig. 4A, Skp2 mRNA expression levels were significantly reduced (P<0.05), whereas c-Myc mRNA expression levels were increased up to ~1.6-fold following exposure to AMP (10, 20 and 30 μM) for 48 h, as compared with the control treatment. c-Myc and Skp2 were also downregulated at the protein level (Fig. 4B). These findings indicate that a proteasomal degradation pathway may be involved in this process. However, as AMP treatment resulted in reduced Skp2 mRNA expression levels, a different proteasome recognition subunit, Fbw7 (including the three isoforms in humans, Fbw7α, -β and -γ), which targets c-Myc, was subsequently analyzed.

### AMP reduces Fbw7α, Fbw7γ and GSK3β expression, and increases Thr58-Myc expression at the protein level

**Figure 5:**
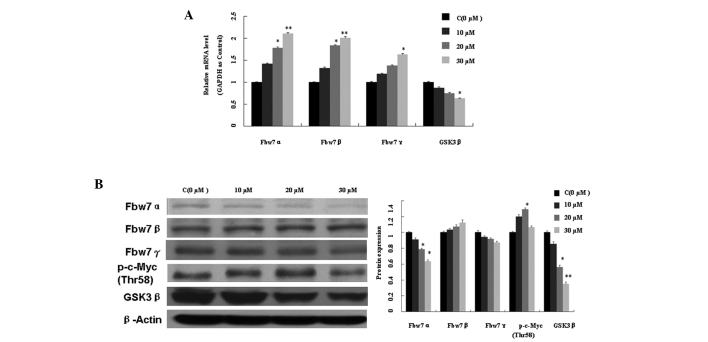
Effects of ampelopsin on Fbw7 and GSK3β expression levels in A549 human pulmonary adenocarcinoma cells. (A) Relative Fbw7α, Fbw7β, Fbw7γ and GSK3β mRNA levels were detected using quantitative polymerase chain reaction with GAPDH as an internal control. The results are expressed as the mean ± standard error of the mean from five independent experiments. (B) Fbw7α, Fbw7β, Fbw7γ, Thr58-Myc and GSK3β protein levels were assayed by western blotting. β-actin served as an internal control.^*^P<0.05 and ^**^P<0.01 vs. the control group. Fbw7, F-box and WD repeat-containing protein 7; GSK3β, glycogen synthase kinase 3β; p−, phosphorylated−.

Following exposure to different concentrations of AMP, the Fbw7α and Fbw7γ mRNA expression levels were increased (Fig. 5A), but with a corresponding reduction in Fbw7α and Fbw7γ protein expression levels (Fig. 5B). Thr58 phosphorylation of c-Myc is a requirement for c-Myc degradation and is mediated by GSK3β. GSK3β is the only kinase that has been previously demonstrated to phosphorylate c-Myc at Thr58 (35,36). However, in the present study, GSK3β expression following AMP treatment was reduced at the mRNA (Fig. 5A) and protein levels (Fig. 5B), as compared with the control treatment, with a certain degree of increase in Thr58 phosphorylation of c-Myc (Fig. 5B). These findings indicate that the phosphorylation of c-Myc at Thr58 may occur independently of GSK3β. The process may therefore involve reduced Fbw7α, Fbw7γ and Skp2 expression levels, but the exact pathway for c-Myc degradation requires further investigation.

## Discussion

AMP is a naturally occurring flavonoid extracted from *Scutellaria baicalensis* Radix, which has been reported to exert antineoplastic activity in various types of cancer (28–34). In the present study, the roles of the c-Myc/Skp2/Fbw7 and HDAC1/2 pathways, which are associated with tumor progression, on the anticancer effects of AMP in the A549 NSCLC line were investigated. The effects of AMP upon A549 cell proliferation and apoptosis were evaluated using MTT assays and Annexin V-PI double staining. The results indicated that AMP inhibited cell growth at half maximal inhibitory concentration <30 μM in a dose-dependent manner. In response to AMP treatment, the early-stage apoptotic rate was increased, since Annexin V-positive cells gradually became Annexin V-negative. Significant apoptosis was observed following 30 μM AMP treatment, as compared with the control treatment, a finding consistent with the morphologic changes observed subsequent to cell nuclear DAPI staining. As shown in Fig. 1D, condensed and cleaved nuclei were detected only in the treatment groups.

To identify the apoptotic effects of AMP treatment at the protein level, the expression levels of apoptosis-related proteins, including Mcl-1, PARP, survivin, Bcl-2 and XIAP were assessed. The results indicated that AMP influenced mitochondrial membrane stability and reduced the mitochondrial membrane potential (Δψm; Fig. 2A). This process may be associated with the reduced Bcl-2 and increased Bax expression levels observed (Fig. 2B). Cleaved PARP and reduced XIAP and survivin expression levels may also have partially contributed to the progression of apoptosis.

HDAC1 and 2, Class I HDACs, not only deacetylate histone/non-histone proteins, but also inhibit gene expression and modify tumor suppressive proteins associated with tumor progression (3,4). The results of the present study revealed that the expression levels of HDAC1/2 were reduced at the mRNA and protein levels in the presence of AMP (Fig. 3), indicating that acetylated histone proteins may promote the expression of tumor suppressive proteins and thus inhibit tumor progression.

A previous study demonstrated that c-Myc, as a transcription factor, has the ability to regulate various genes involved in cellular proliferation, differentiation, growth and apoptosis (7). The post-translational regulation of c-Myc is mediated by Skp2 and Fbw7 (12). The c-Myc and Skp2 oncoproteins are inter-related such that c-Myc promotes Skp2 expression and Skp2 targets c-Myc for ubiquitin-dependent degradation. In the present study, AMP downregulated Skp2 expression at the mRNA level and protein levels. In addition, AMP downregulated c-Myc at the protein level, but c-Myc mRNA expression levels were increased (Fig. 4). These findings suggest that the proteasomal degradation pathway may be associated with the reversal of c-Myc. However, since Skp2 expression levels were reduced, subsequent experiments were focused on Fbw7 (which has three isoforms in humans: Fbw7α, β and γ), another proteasome recognition subunit that targets c-Myc.

The involvement of Fbw7 deficiency in drug resistance in human cancer has been recently identified (22,23). In the regulation of c-Myc, Fbw7 is different from Skp2. Thr58 phosphorylation of c-Myc is required for Fbw7α, β and γ, which have been shown to mediate c-Myc ubiquitin-dependent degradation (25), and GSK3β is the only known kinase that phosphorylates c-Myc at Thr58 (24). In the present study, the expression levels of Fbw7α, Fbw7β, Fbw7γ, p-c-Myc (Thr58) and GSK3β were evaluated.

Fbw7α and Fbw7γ expression following AMP treatment were found to be reduced at the protein level, but increased at the mRNA level (Fig. 5). Thr58 phosphorylation of c-Myc was increased to a certain degree, and GSK3β expression at the mRNA and protein levels was reduced. These results highlight the lack of conformity between Fbw7α/γ mRNA and protein levels, the reduced expression levels of GSK3β and the increased phosphorylation of c-Myc at Thr58. These findings suggest that AMP induces apoptosis independently of Fbwα/γ and that the phosphorylation of c-Myc at Thr58 may occur independently of GSK3β, a finding concurrent with those of previous studies that observed phosphorylation of Thr58 in A549 cells occurring independently of GSK3β (37,38).

In conclusion, the results of the present study indicate that AMP influences multiple biochemical pathways, which may explain AMP activity against various types of cancer. In addition, the results may also partially explain why cells deficient in certain genes, for instance Fbw7-deficient cells, are resistant to AMP and suggest a possible use of AMP in drug-resistant cancer associated with Fbw7 deficiency. However, the exact underlying mechanism of AMP action, which pathways are associated with c-Myc and Fbw7α/γ reversal and whether Thr58 phosphorylation of c-Myc is dependent on GSK3β requires further investigation.

## Figures and Tables

**Table I:** Primer sequences for quantitative polymerase chain reaction.

Gene	Orientation	Primer sequence (5′-3′)
GAPDH	Forward	GAAATCCCATCACCATCTTCCAGG
	Reverse	GAGCCCCAGCCTTCTCCATG
HDAC1	Forward	TAAATTCTTGCGCTCCATCC
	Reverse	AACAGGCCATCGAATACTGG
HDAC2	Forward	CGTGTAATGACGGTATCATTCC
	Reverse	ACCAGATAATGAGTCTGCACC
c-Myc	Forward	AGCGACTCTGAGGAGGAACAAG
	Reverse	GTGGCACCTCTTGAGGACCA
Skp2	Forward	TGGGAATCTTTTCCTGTCTG
	Reverse	GAACACTGAGACAGTATGCC
Fbw7α	Forward	AGTAGTATTGTGGACCTGCCCGTT
	Reverse	GACCTCAGAACCATGGTCCAACTT
GSK3β	Forward	GGCAGCATGAAAGTTAGCAGA
	Reverse	GGCGACCAGTTCTCCTGAATC

HDAC, histone deacetylase; Skp2, S-phase kinase-associated protein 2; Fbw7α, F-box and WD repeat-containing protein 7α; GSK3β, glycogen synthase kinase 3β.
